# Comparative MD Simulations Indicate a Dual Role for Arg132^3.50^ in Dopamine-Dependent D2R Activation

**DOI:** 10.1371/journal.pone.0146612

**Published:** 2016-01-07

**Authors:** Ralf C. Kling, Timothy Clark, Peter Gmeiner

**Affiliations:** 1 Department of Chemistry and Pharmacy, Emil Fischer Center, Friedrich Alexander University, Erlangen, Germany; 2 Department of Chemistry and Pharmacy, Computer Chemistry Center, Friedrich Alexander University, Erlangen, Germany; 3 Centre for Molecular Design, University of Portsmouth, King Henry Building, Portsmouth, United Kingdom; University of Connecticut, UNITED STATES

## Abstract

Residue Arg^3.50^ belongs to the highly conserved DRY-motif of class A GPCRs, which is located at the bottom of TM3. On the one hand, Arg^3.50^ has been reported to help stabilize the inactive state of GPCRs, but on the other hand has also been shown to be crucial for stabilizing active receptor conformations and mediating receptor-G protein coupling. The combined results of these studies suggest that the exact function of Arg^3.50^ is likely to be receptor-dependent and must be characterized independently for every GPCR. Consequently, we now present comparative molecular-dynamics simulations that use our recently described inactive-state and Gα-bound active-state homology models of the dopamine D2 receptor (D2R), which are either bound to dopamine or ligand-free, performed to identify the function of Arg132^3.50^ in D2R. Our results are consistent with a dynamic model of D2R activation in which Arg132^3.50^ adopts a dual role, both by stabilizing the inactive-state receptor conformation and enhancing dopamine-dependent D2R-G protein coupling.

## Introduction

Residue Arg^3.50^ (the superscript refers to the generic Ballesteros-Weinstein numbering[1]) belongs to the DRY-motif of G protein-coupled receptors (GPCRs), which is located at the base of transmembrane helix (TM) 3, and had been suggested to play a vital role in regulating the structure and function of these receptors.[2] The individual residues of the DRY-motif, aspartate (or glutamate), arginine and tyrosine, are highly conserved among class A GPCRs; their degree of conservation is 67.9% (D)/22.8% (E), 96.8% and 71.5%, respectively (calculated by means of the GMOS web interface, http://lmc.uab.cat/gmos/cgmos.php).

Arg^3.50^ is part of the so-called ionic lock, an intramolecular ionic interaction between Arg^3.50^ of TM3 and Glu^6.30^ of TM6, which was originally observed in the crystal structure of dark-state rhodopsin.[3] As alanine mutation of Glu^6.30^ has been shown to enhance constitutive activity at the β2 adrenergic receptor (β2AR), the ionic lock was suggested to help stabilize the ground state of GPCRs.[4, 5] With the exception of rhodopsin,[3] the dopamine D3 receptor (D3R) [6] and distinct β1 adrenergic receptor (β1AR)-ligand combinations,[7] an intact ionic lock is not observed within the majority of currently available crystal structures. However, several independently performed molecular-dynamics (MD) simulations have reported the highly dynamic nature of this interaction, with an equilibrium between intact and broken ionic-lock conformations, which is likely to reflect the basal activity of non-rhodopsin GPCRs.[5, 8–10] The existence of open and closed states of the ionic lock, connected to different conformations of TM6, had been supported by crystal structures of β1AR.[7] Nevertheless, only 34% (calculated by means of the GMOS web interface, http://lmc.uab.cat/gmos/cgmos.php) of class A GPCRs exhibit both an arginine and a glutamate residue at positions 3.50 of TM3 and 6.30 of TM6, respectively. This suggests that an intact ionic lock may not be the only determinant that stabilizes inactive-state GPCRs. Thus, it was not possible to reduce the pronounced constitutive activity of the wild-type histamine H4 receptor (H4R), which has an alanine residue in position 6.30, when trying to reconstitute the possibility of forming an ionic interaction to Arg^3.50^ using an Ala^6.30^Glu mutant receptor.[11]

Besides its (possible) contribution to the basal signaling profile of GPCRs, the crystal structure of opsin in complex with the C-terminal fragment of transducin revealed hydrogen bonds between the side chain of Arg^3.50^ and that of Tyr^5.58^ of TM5. Arg^3.50^ also hydrogen bonds to the backbone carbonyl atom of Cys347 of the G protein, thus attributing a key role to Arg^3.50^ in stabilizing active-state GPCR conformations and mediating receptor-G protein interactions.[12] Based on this structure, we recently performed computational studies on β2AR together with the C-terminal fragment of Gα_s_, in which direct interactions between Arg^3.50^ and residues of the G protein could be observed.[13] The crystal structure of β2AR coupled to the heterotrimeric Gs protein confirmed such direct interactions: the side chain of Arg^3.50^ was found to pack against Tyr391 of Gα_s_.[14] In addition, MD simulations on our previously developed homology model of the dopamine D2 receptor (D2R)-Gα_i_ complex indicated an ionic interaction of Arg132^3.50^ and a C-terminal residue Asp350 of Gα_i_.[15] This ionic interaction was found to persist in the presence of the full agonist dopamine, but to be destabilized by aripiprazole-type partial agonists.[16] In agreement with these studies, different groups have reported reduced or abolished G-protein activation when Arg^3.50^ of wild-type receptors is mutated to alanine, including D2R,[17] rhodopsin[18] and H4R.[11] In addition, it was shown that mutations of Arg^3.50^ that cause a loss of the capacity to couple to or to activate G proteins can culminate in diseases such as autosomal dominant retinitis pigmentosa (ADRP),[18] nephrogenic diabetes insipidus[19] or hypogonadotropic hypogonadism.[20] However, no unified picture of the influence of Arg^3.50^ on G protein activation can be generated, as, for example, different mutations of Arg^3.50^ at β2AR were connected to an unchanged ability to activate Gα_s_ (even if the capacity to recruit β-arrestin was reduced for the Arg^3.50^Ala mutant).[5, 21] A more detailed discussion of the effect of distinct Arg^3.50^ mutations at different receptors is provided in the literature.[2]

Taken together, these results suggest that the exact function of Arg^3.50^ is likely to be receptor-dependent and must be characterized independently for every GPCR. As (I) D2R exhibits both residues Arg132^3.50^ and Glu368^6.30^, and is thus, in principal, competent to form an ionic lock interaction, and (II) previous studies on the (dopamine-bound) D2R-Gα_i_ complex suggested a direct ionic interaction between Arg132^3.50^ and the G protein,[15, 16] we chose to investigate these interactions for inactive- and active-state D2R conformations. Therefore, while taking advantage of the recent developments in the structural determination of GPCRs, a comparative analysis of MD simulations that use our inactive-state[22] and active-state homology models of D2R,[15] both bound to dopamine or ligand-free (apo), was performed to identify the function of Arg132^3.50^ at D2R.

## Results/Discussion

### Stability of the Simulation Systems

**Fig 1:**
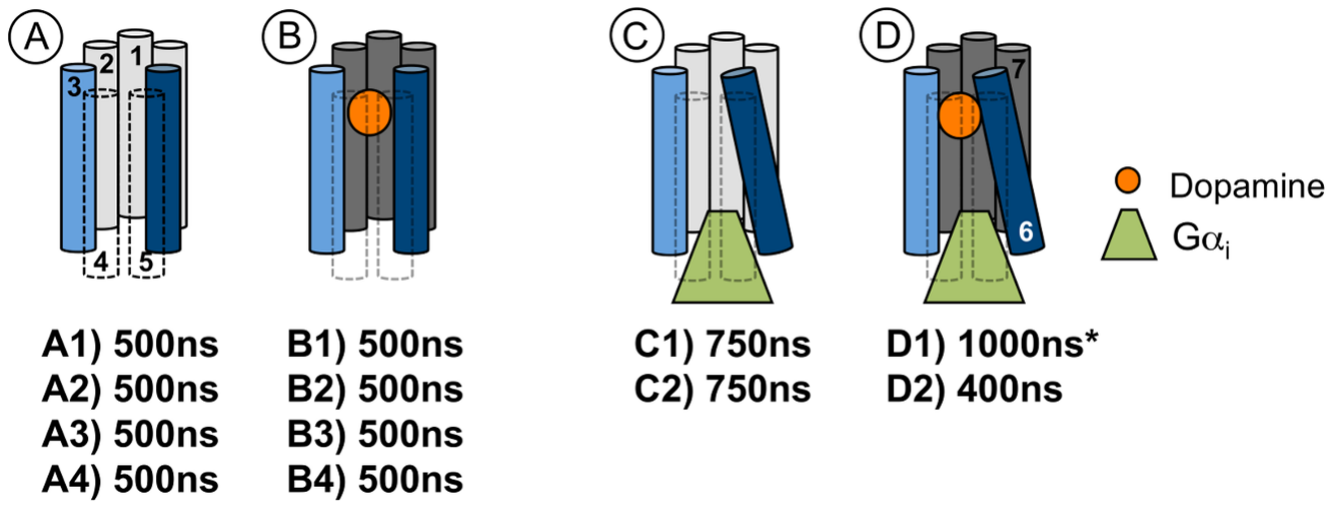
Schematic overview of the main simulation systems and their simulation times. To visually help distinguish the apo- (in which dopamine is absent, A and C) from the dopamine-bound complexes (dopamine in orange, B and D), TM 1, 2 and 7 are colored in light-grey and dark-grey, respectively. For clarity, TM3 and TM6 are colored in light-blue and dark-blue, respectively. The active-state systems (C and D) are represented by the characteristic outward movement of TM6 and the presence of Gα_i_ (in green). The asterisk refers to a previously published simulation. The simulation times of each system are given in bold.

**Fig 2:**
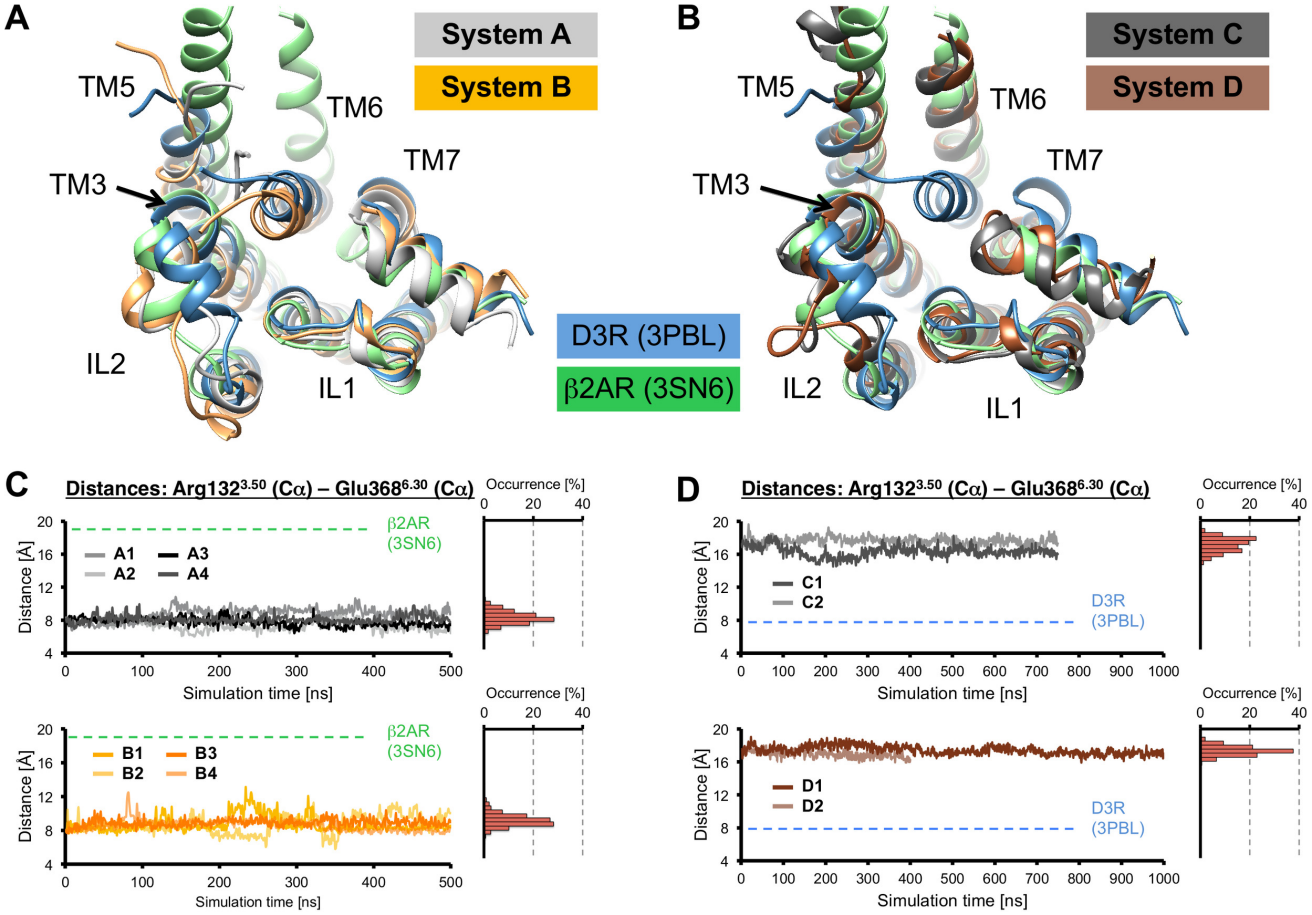
Conformational classification of the simulation systems A-D. (A, B) Intracellular view on cytoplasmic receptor domains of the simulations systems A-D indicating unchanged global conformational states throughout the MD simulations: An overlay of average structures of representative simulation systems (each derived from the final 25ns simulation times) is shown for (A) inactive-state systems A (light-grey) and B (light-orange) and (B) the active-state systems C (dark-grey) and D (brown). For comparison, the X-ray structures of inactive-state D3R (blue) and active-state β2AR (green), which were used for homology modeling, are depicted. (C, D) The distances (and occurrences of these distances) between the intracellular ends of TM3 and TM6, measured as the distances between the Cα-atoms of Arg132^3.50^ and Glu368^6.30^, are shown for (C) the inactive-state systems A and B and (D) the active-state systems C and D. For comparison, these distances at crystal structures of D3R and β2AR are highlighted with dashed lines. Colors are used as described above.

Eleven individual long-term MD simulations were performed on homology models of D2R, which were either coupled to dopamine, (and/or) Gα_i_ or did not contain an additional binding partner (Fig 1). Data derived from previous MD simulations on a dopamine-bound D2R-Gα_i_ complex were used for comparison (system D1). The overall conformational stability of the different complexes was found to be sufficient for subsequent analyses, as indicated by RMSD analysis of the individual members of the simulation systems (S1 Fig), which did not undergo destructive conformational changes that affected the integrity of the complexes. Within the Gα_i_-bound systems (complexes C1, C2 and D2), higher mobility was observed for Gα_i_ than for the receptor (in particular, the helical subdomain of the Gα_i_-subunit (Gα_i_AH), S2 Fig), which is in agreement with our previous studies on ternary complexes,[15, 16] and, as previously, Gα_i_ did not show any tendency to separate from active-state D2R (S3 Fig). Importantly, the global conformational state of the receptors (either inactive- or active-state like) did not change throughout the simulation time, as determined by measuring the distances between the intracellular tips of TM3 and TM6 (Fig 2). In the course of this study, the active-state of D2R is characterized by the outward movement of TM6 and the presence of the Gα_i_-subunit of the G protein (systems C and D), whereas the inactive-state systems lack the latter features (systems A and B). Visual comparison of several overlaid average structures of systems A-D derived from different time windows along the simulation pathways indicated that the presence of dopamine in the systems B and D was associated with a reduced mobility of extracellular receptor domains compared to the apo-simulations. This stabilizing effect was significantly more pronounced in the active-state simulations C and D (S4 Fig). Moreover, the presence of dopamine was found to increase the conformational stability of the outward movement of TM6 in the absence of the G protein (S5 Fig).

### Analysis of Dopamine Binding at Inactive- and Active-State D2R Models

**Fig 3:**
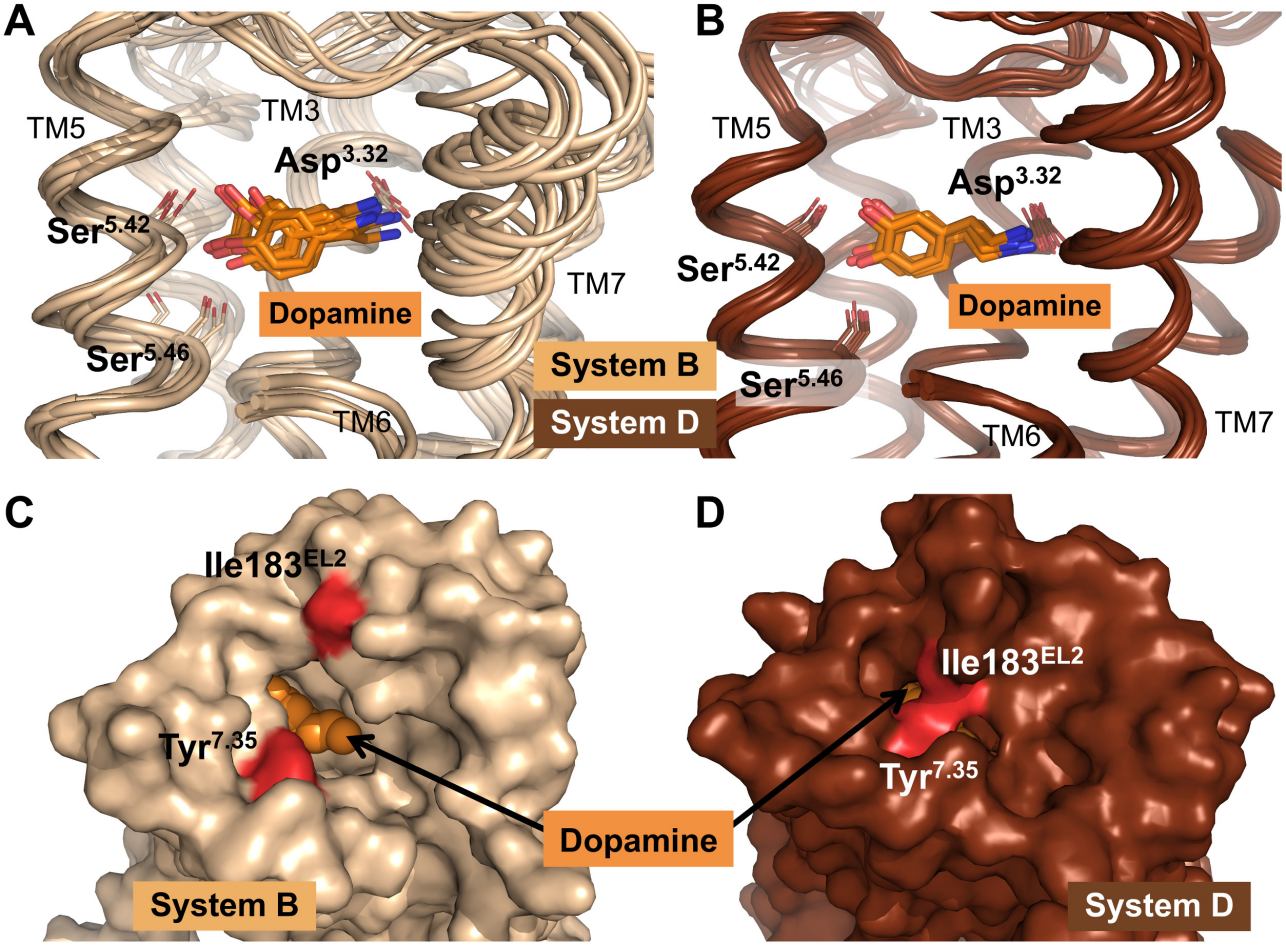
Overview of the binding pockets of dopamine at the simulations systems B and D. (A, B) Side view of representative average structures derived from different time points along the MD simulation pathways, focusing on the binding pockets of dopamine (orange) at inactive-state (system B, ocher) and active-state (system D, brown) D2R. TM6 has been removed for clarity. In each complex, dopamine is stabilized by hydrogen bonds to Ser193^5.42^ and Ser197^5.46^ and by an ionic interaction to Asp114^3.32^, but shows a slightly higher mobility at inactive-state D2R (system B). (C, D) Top view onto the binding pockets of representative snapshots of the systems B1 (ocher) and D1 (brown). Each structure is derived from the final 25ns of simulation time. Dopamine is shown as orange spheres. In system B1, the binding pocket is open to the extracellular surface of D2R, showing a large distance between Ile183^EL2^ and Tyr408^7.35^ (indicated in red), whereas the binding pocket in system D1 is closed to the extracellular surface by these residues approaching each other.

Within the dopamine-bound systems B and D, dopamine was found to occupy, as expected, the same orthosteric binding pocket throughout the MD simulations in both the inactive- and active-state D2R, and to adopt a similar conformation therein (Fig 3A and 3B). The conformation of dopamine is stabilized by hydrogen bonds between its catechol moiety and Ser193^5.42^ and Ser197^5.46^ of TM5 and His393^6.55^ of TM6 (not shown), all of which are in agreement with previous studies reporting their importance for the binding of dopamine.[23, 24] In addition, the canonical salt bridge between the protonated amine moiety of dopamine and Asp114^3.32^ of TM3 was formed persistently, an interaction that has been shown to be an irreplaceable prerequisite for specific ligand binding at dopaminergic receptors.[25] Although the presence of Gα_i_ did not significantly alter the nature and occurrence of intermolecular interactions between dopamine and D2R relative to inactive-state D2R, a slightly reduced dopamine mobility and an increase of 4.8 kcal/mol in its binding energy were observed (Fig 3A and 3B, S6 Fig). These observations are obviously the consequence of different shapes of the extracellular surface above the binding pocket of D2R (measured as the distance between Ile183 of extracellular loop 2 (EL2) and Tyr408^7.35^ of TM7, S7 Fig). A persistently closed conformation around the agonist dopamine was found in simulations of the fully active ternary signaling complex, thus facilitating the stabilization of dopamine (Fig 3C and 3D). A closed structure above the binding pocket of dopamine was originally observed in previous simulations of system D1,[16] and could now be confirmed by an additional MD simulation (system D2). Increased distances between EL2 and the upper part of TM7 associated with an open binding pocket to the extracellular surface were observed in both apo-D2R simulations (S4 and S7 Figs). The observation that neither the presence of dopamine (system B) nor of Gα_i_ alone (system C, representing the basally-active signaling state of D2R) were sufficient to result in a persistent and stable contraction of extracellular domains near the binding pocket supports observations that both agonists and an intracellular binding partner are required to capture fully active-state conformations of GPCRs,[26, 27] including those of active-state binding pockets. However, the possibility that such contractions would eventually be triggered on much longer time-scales cannot be excluded.

### Dopamine-Binding at Inactive-State D2R Reduces the Stability of the Ionic Lock

The overall aim of this study was to identify the function of Arg132^3.50^ in the pharmacologically relevant D2R, which includes in particular the investigation whether or not an intramolecular ionic interaction to Glu368^6.30^ at the inactive-state receptor can be formed, thus stabilizing the ground-state of D2R, and whether this ionic interaction can be modulated by the presence of the endogenous agonist dopamine. Consequently, this chapter will focus on the analysis of 4 μs MD simulations at inactive-state D2R, which were performed under two different conditions: ligand-free (apo, system A) or bound to dopamine (system B).

**Fig 4:**
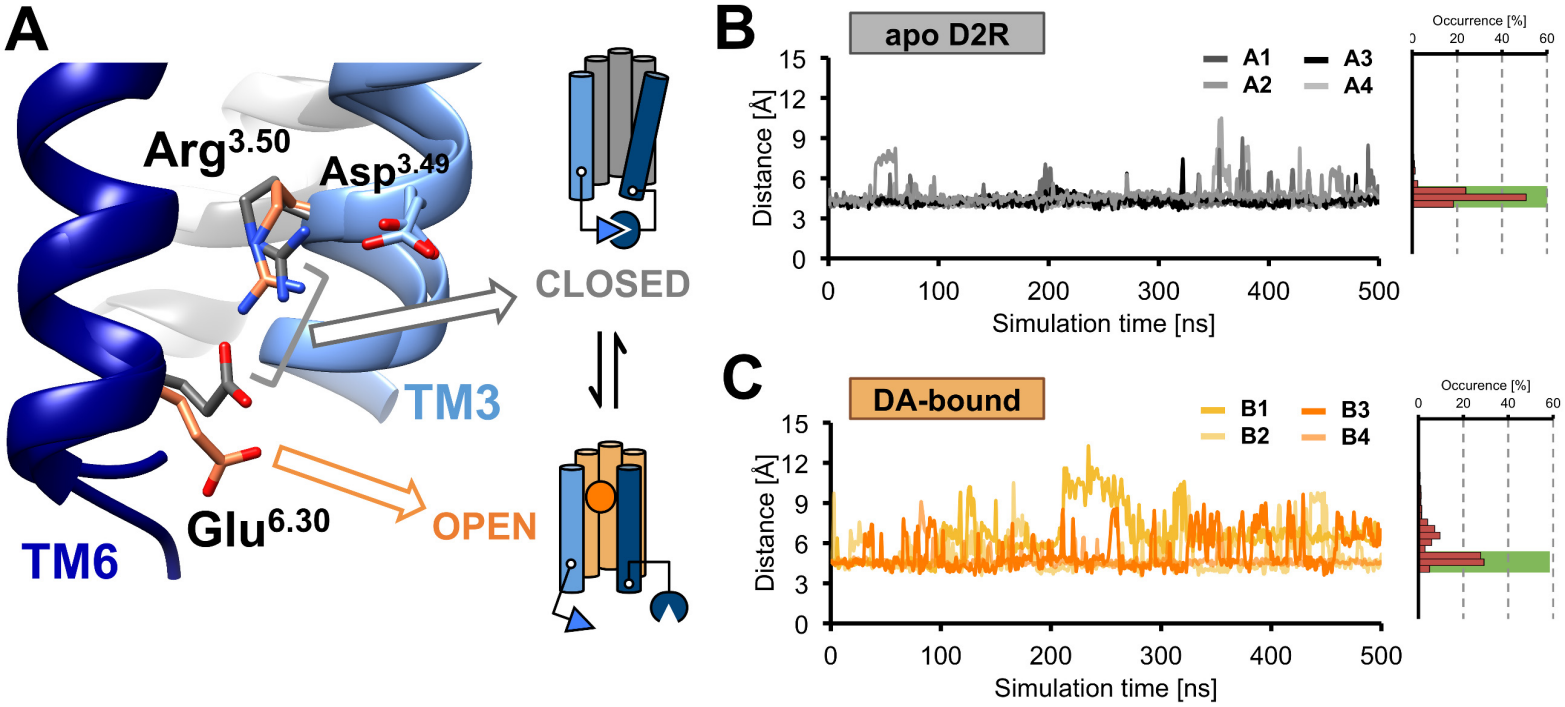
Distances of ionic lock residues at the inactive-state systems A and B. (A) Close view on representative conformations of an intact (= closed, grey) and a broken (= open, orange) ionic lock between residues Arg132^3.50^ of TM3 (light-blue) and Glu368^6.30^ of TM6 (dark-blue). In addition, Arg132^3.50^ is stabilized by Asp131^3.49^ of TM3. (B, C) Distances between the side chains of residues Arg132^3.50^ (Cζ) and Glu368^6.30^ (Cδ) in the course of the simulations A and B are shown. Cumulative occurrences of certain distances for system A (B) predominantly show distances, which are consistent with an intact ionic lock (green boxes). In contrast, the latter distances are less frequently populated at the dopamine-bound system B (C), when higher occurrences were observed for larger distances, consistent with an open ionic lock.

**Fig 5:**
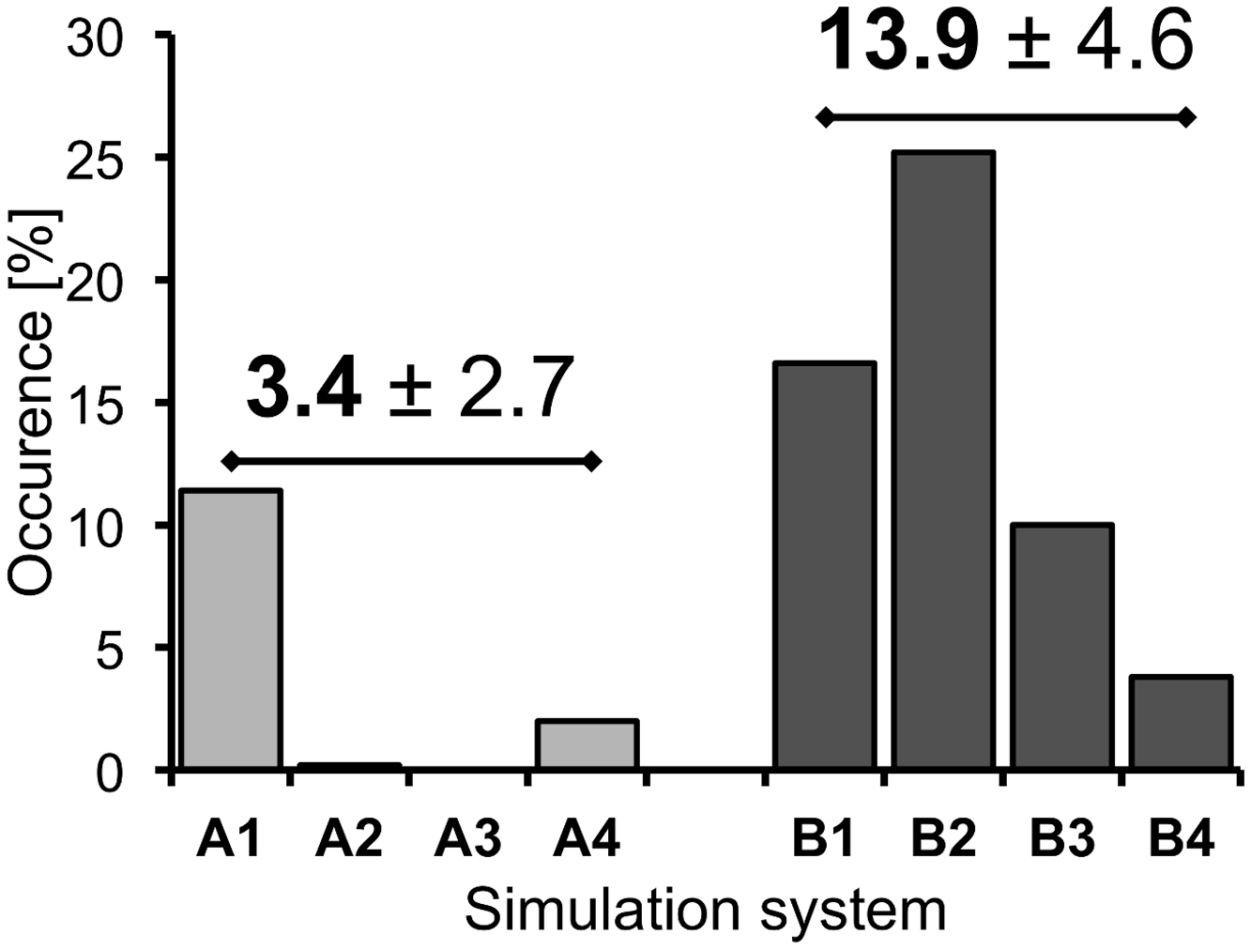
Total occurrences of distances larger than 9.5 Å between Arg132^3.50^ and Glu368^6.30^ at the simulation systems A and B. The fractions of simulation time within the systems A and B, in which the distances between the Cα-atoms of Arg132^3.50^ and Glu368^6.30^ were found to be larger than 9.5 Å. The values above the bars represent mean ± standard error of the mean of the simulation systems A and B and indicate a higher frequency of distances larger than 9.5 Å in the presence of dopamine (unpaired t-test, two-tailed P value = 0.0960).

In general, our results indicate that the inactive-state of D2R is able to adopt both formed (= closed) and broken (= open) conformations of the ionic lock, which were found to exist in dynamic equilibrium with each other (Fig 4A). These observations are in excellent agreement with previous MD simulations on closely related adrenergic receptors, which had reported alternately open and closed conformations of the ionic lock.[5, 8, 9] At β1AR, it was even possible to crystallize the different states of this motif, where such structural plasticity of intracellular receptor domains had been suggested to be a general feature of non-rhodopsin GPCRs, which exhibit varying capacities for ligand-independent signaling (also referred to as basal activity).[7] The evolution of side-chain distances between Arg132^3.50^ (Cζ) of TM3 and Glu368^6.30^ (Cδ) of TM6 revealed that in MD simulations of the apo D2R-system A (representing the ligand-free ground state of D2R), the formation of an intact ionic lock between these residues is highly favored (Fig 4B). Thus, in the absence of an agonist, the ionic lock was closed most of the time, which is likely to help stabilize the inactive, ground state conformation of D2R. The latter assumption is supported by previous MD simulations studies on both carazolol-bound and apo β2AR, demonstrating that the extent of ionic-lock formation in the presence of the inverse agonist carazolol, which is known to stabilize the inactive-state of β2AR, is unchanged when compared to the ligand-free β2AR system.[8] In contrast, we observed that the presence of the endogenous agonist dopamine (system B) significantly reduced the occurrence of an intact ionic lock (Fig 4C), which is likely to result in an impaired capacity of this intramolecular interaction to stabilize the inactive-state of D2R. A comparable, agonist-dependent decreasing effect on the frequency of ionic-lock conformations was also suggested by MD simulations at the 5-HT_2A_ receptor.[10] In addition to the enhanced probability of encountering a broken ionic lock, we found that in the presence of dopamine, conformations featuring distances larger than 9.5 Å between the intracellular ends of TM3 and TM6 (measured as the Cα-distance of Arg132^3.50^ and Glu368^6.30^, Fig 2), were increased compared to the ligand-free system A (Fig 5). Distances larger than 9.5 Å were previously shown to be associated with broken ionic lock conformations by MD simulations and crystal structures.[8, 9] The separation of TM3 and TM6 is associated with an outward movement of TM6, which represents a major hallmark of GPCR activation.[28] However, additional simulations are needed to increase the statistical significance of the result shown in Fig 5.

Taken together, our results are consistent with a structural mechanism of dopamine-dependent D2R activation, by which the agonist dopamine reduces the stability of the ionic lock, thereby reducing the global conformational stability of the inactive-state of D2R, and thus increases the probability for an outward movement of TM6, which finally facilitates receptor activation. It is important to note that even in the absence of dopamine (system A), we detected open ionic lock conformations and a minor fraction of TM6 showing limited outward movement, both of which are consistent with the basal activity profile of D2R. Although we are aware that there may also be other intramolecular interactions that stabilize the inactive-state of D2R, it is tempting to assume that the breakage of the ionic lock is one crucial and necessary prerequisite in the activation process of D2R.

### Dopamine Binding at Active-State D2R-Gα_i_ Complexes Triggers the Formation of an Ionic Interaction between D2R and Gα_i_ via Arg132^3.50^

Crystal structures of opsin and β2AR coupled to the C-terminal fragment of transducin and the natural Gs protein, respectively, revealed direct interactions of receptors and G proteins, which were, among others, mediated by residue Arg^3.50^ of the receptors.[12, 14] The structures supported various experiments employing Arg^3.50^ receptor mutants, which had attributed a key role to this residue in maintaining the active-state of a GPCR.[2] In addition, by using long-term MD simulations on a dopamine-bound ternary D2R-Gα_i_ complex model based on the crystal structure of β2AR coupled to Gα_s_,[15] we previously detected a consistent ionic interaction between Arg132^3.50^ of D2R and Asp350 of Gα_i_, which we suggested to help stabilize receptor-G protein coupling.[16] The latter observation is supported by experiments using an Arg132^3.50^Ala mutant of D2R, which completely lost the capacity to activate G proteins upon agonist stimulation.[17] As the final part of this study, which was designed to investigate the function of Arg132^3.50^ in both inactive- and active-state conformations of D2R, we now focus on the analysis of the structural properties of this latter ionic interaction, employing a total of 3.9 μs MD simulations performed on either dopamine-bound (system D) or ligand-free (system C) D2R-Gα_i_ models.

**Fig 6:**
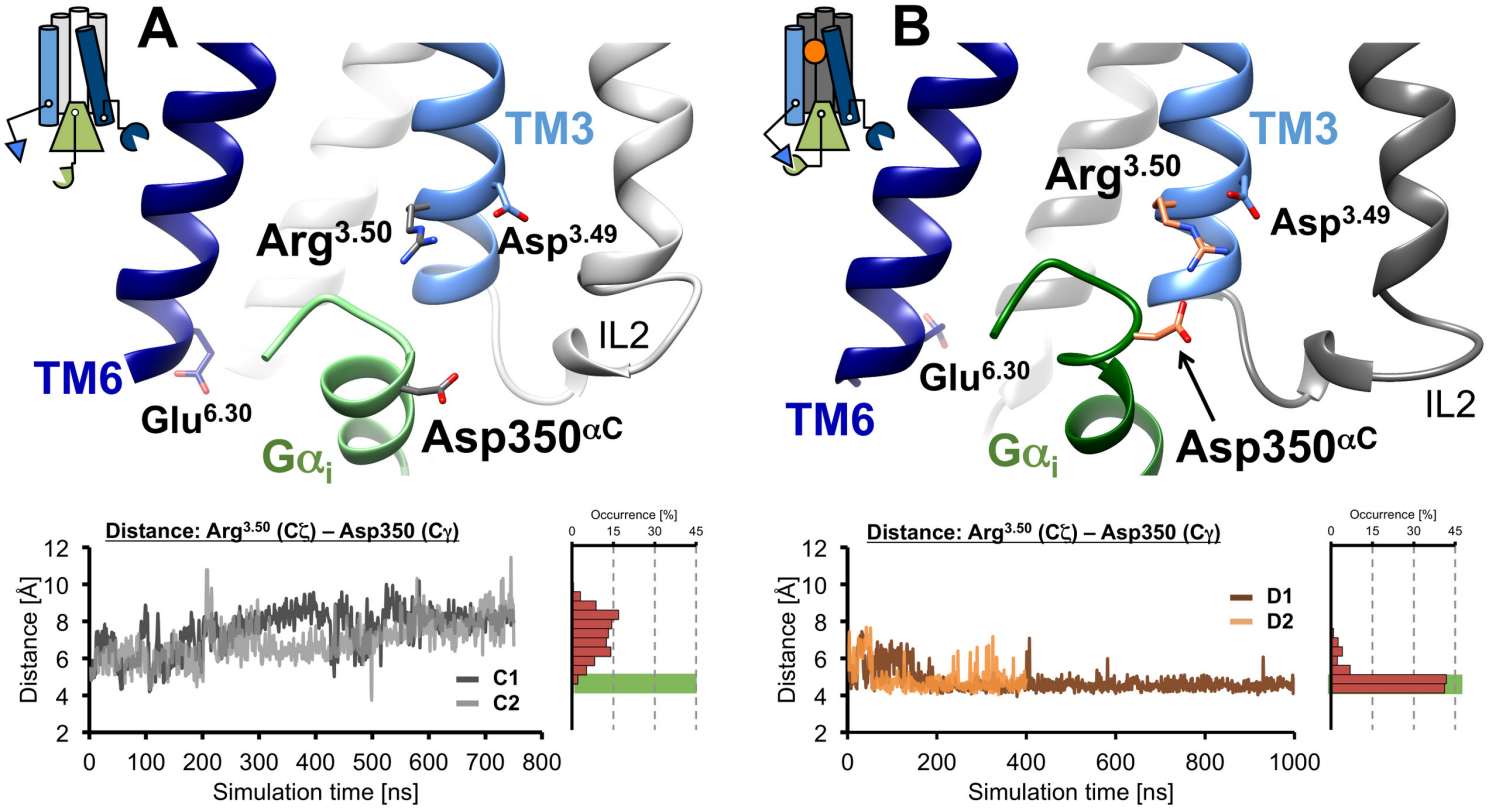
Time evolution of the ionic interaction between D2R and Gα_i_ at the active-state systems C and D. Representative snapshots of ligand-free (A, top line) and dopamine-bound (B, top line) active-state D2R conformations, showing an either broken or formed ionic interaction between residues Arg132^3.50^ of D2R and Asp350 of Gα_i_, respectively. In addition, the time evolution of distances between Arg132^3.50^ (Cζ) of D2R and Asp350 (Cγ) of Gα_i_ in simulation systems C (A, bottom line) and D (B, bottom line) are depicted, revealing that distances, which allow the ionic interaction between D2R and Gα_i_ (green boxes) are only formed in the presence of dopamine.

We detected the formation of a consistent ionic interaction between Arg132^3.50^ of D2R and Asp350 of Gα_i_ after approximately 200 ns in the presence of dopamine. This was further corroborated by an additional MD simulation run on the same dopamine-bound ternary D2R-Gα_i_ complex (system D2), in which this ionic interaction was formed reproducibly and remained, in both cases, stable for most of the simulation time (Fig 6B). In the absence of dopamine (system C), increasing distances between the corresponding residues Arg132^3.50^ of D2R and Asp350 of Gα_i_ were observed in two independent simulation runs on the same apo D2R-Gα_i_ complex, indicating that the aforementioned ionic interaction can hardly be formed in the ligand-free, basally active-state of D2R (Fig 6A). It is thus tempting to speculate that Arg132^3.50^ may play a crucial role in mediating a ligand-induced increase in G protein activation.

Interestingly, these results show that a valid answer whether or not an ionic interaction between Arg132^3.50^ and Asp350 can be formed in the particular simulation systems C1 and C2 does not become evident before a certain “induction period”, in these large systems of more than 200,000 atoms at least 200 ns (Fig 6). This is in line with ten individual MD simulations on system C (systems C3 to C12), each using the same configuration than C1 and C2, randomly attributed initial velocities and each lasting 100 ns, which do not show a clear tendency of the system to form an ionic interaction between Arg132^3.50^ and Asp350 (S8 Fig). These observations support previous studies,[13, 15, 16, 29] in which our group has performed few long simulations, rather than multiple shorter ones, in order “*to avoid missing conformational changes that occur with a characteristic induction period*”.[16]

In summary, our analyses of MD simulations on the active-state systems C and D demonstrate that the ionic interaction between D2R and Gα_i_ strongly depends on the presence of an agonist like dopamine. Only in the presence of dopamine (system D, Fig 6B) does this interaction remain stable throughout the simulation time. We previously observed a reduced stability of this particular ionic interaction once the full agonist dopamine was replaced by aripiprazole-type partial agonists.[16] As agonists are known to enhance the capacity of GPCRs to activate G proteins according to their distinct intrinsic efficacies,[30] we suggest that, at least in the case of the D2R- Gα_i_ complex, an agonist-dependent increase in the capacity of D2R to form an ionic interaction to Gα_i_ via Arg132^3.50^ provides one structural explanation for the question as to how this enhanced activation can be accomplished.

## Conclusion

To identify the function of Arg132^3.50^ at D2R in terms of forming the intramolecular ionic lock between TM3 and TM6 and an ionic interaction to the G protein, comparative MD simulations on both inactive-state and Gα-bound active-state D2R models, which were either ligand-free or bound to the endogenous agonist dopamine (Fig 1), were used.

Within the dopamine-bound systems B and D, our MD simulations detected different conformations of the extracellular surface above the binding pocket adopting a closed conformation in the fully activated Gα-bound system D (Fig 3), although the interactions of dopamine with residues of D2R were comparable for active- and inactive-state D2R. As a result of MD simulations on the inactive-state of D2R, we found that dopamine was able to attenuate ionic-lock formation between Arg132^3.50^ of TM3 and Glu368^6.30^ of TM6 compared to the ligand-free system (Fig 4). As a consequence, higher occupancies of increased distances between the intracellular ends of TM3 and TM6 were recorded in the presence of dopamine, which are consistent with a more pronounced outward movement of TM6 (Fig 5). Both of these observations are in full agreement with the ability of dopamine to trigger D2R activation. Conducting MD simulations that used the D2R-Gα_i_ complexes, we observed a consistent ionic interaction between Arg132^3.50^ of D2R and Asp350 of Gα_i_ in the presence of dopamine, which was not formed within the apo simulation (Fig 6), and which we believe is one structural explanation for an enhanced G protein activation of agonist-bound D2R.

**Fig 7:**
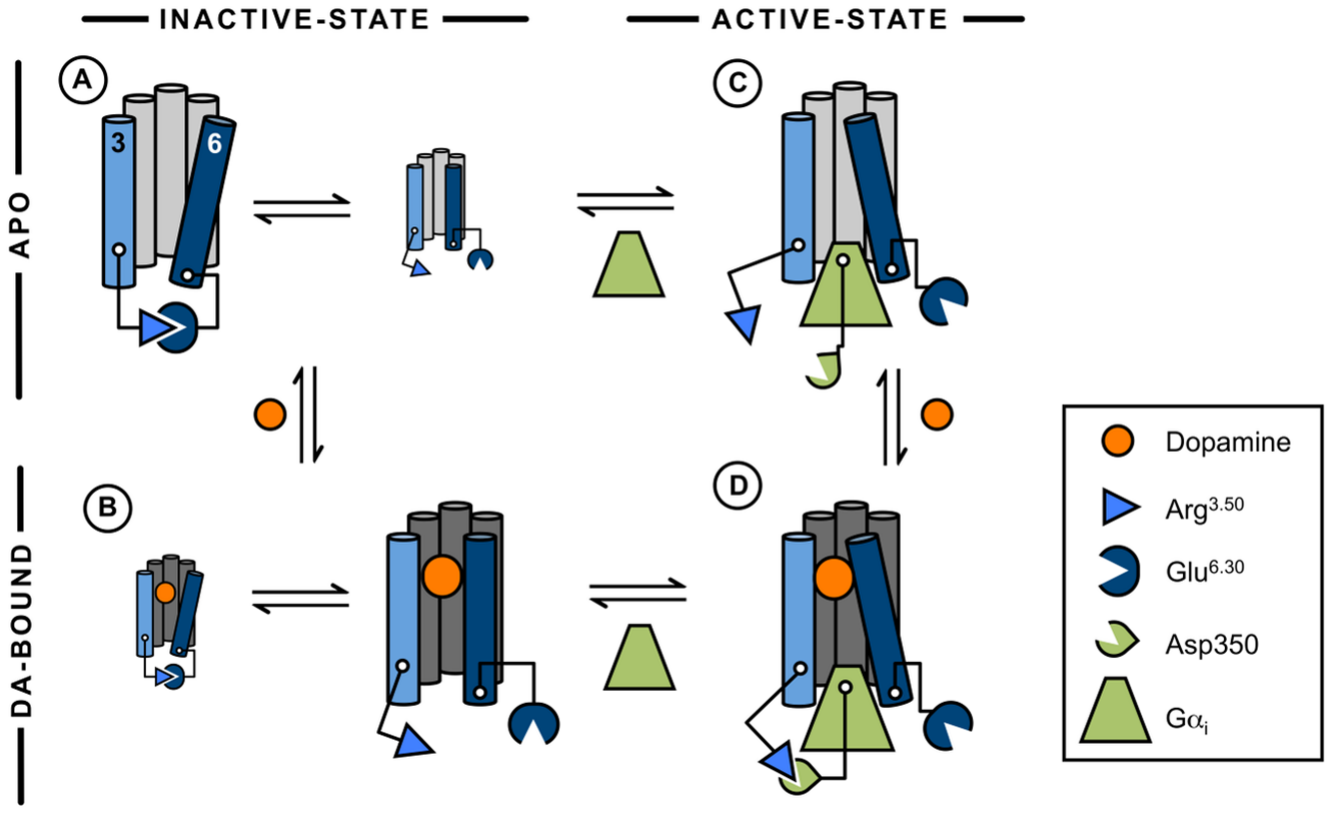
Concluding model of the predicted impact of Arg132^3.50^ on dopamine-dependent activation of D2R. At the inactive-state system (left row), an equilibrium between a formed and a broken ionic lock was observed, whereas the presence of dopamine was found to reduce the stability of the ionic lock. At active-state D2R (right row), dopamine increased receptor-G protein interactions via the formation of an ionic interaction between Arg132^3.50^ of D2R and Asp350 of Gα_i_. For clarity, TM3 and TM6 are colored in light-blue and dark-blue, respectively.

Taken together, our results lead to a model of D2R activation, in which Arg132^3.50^ participates in this process by adopting a dual role, both by the stabilization of the inactive-state receptor conformation and by enhancing dopamine-dependent D2R-G protein coupling (Fig 7). Although it is still not possible to capture the activation process of GPCRs in a single trajectory using classical MD simulations, this study provides a firm, dynamic model for dopamine-dependent D2R activation.

## Materials and Methods

A schematic overview of the simulation systems and their simulation times is shown in S1 Table. In general, comparative MD simulations on either dopamine-bound or ligand-free homology models of inactive-state D2R and of the active-state D2R-Gα_i1_ complex, which were based on the crystal structures of D3R[6] (PDB-ID: 3PBL) and β2AR-Gα_s_[14] (PDB-ID: 3SN6), respectively, were performed. The homology models were generated as described previously for inactive-state D2R[22] and the active-state complex.[15] Docking of dopamine was performed manually as described.[15] The systems A, B and C were submitted to twenty independent MD simulation runs ranging from 100 ns to 750 ns, and complemented by one additional simulation run of 400 ns for complex D2 (Fig 1). The systems E and F were generated by removing Gα_i_ from the final snapshots of the simulation systems C1 and D2, respectively. Subsequently, two independent simulation runs for system E (850 ns and 700 ns) and F (1050 ns and 1100 ns) were performed. All systems were embedded in a lipid bilayer consisting of dioleoylphosphatidylcholine (DOPC) molecules as described.[15, 31] For comparison, the results of previously published long-term MD simulations on the D2R-Gα_i_ complex in presence of the full agonist dopamine were used (simulation D1).[15, 16]

To carry out MD simulations, the GROMACS simulation package was used as described previously.[13] Briefly, the general AMBER force field (GAFF)[32] was used for dopamine and the lipids and the AMBER force field ff99SB[33] for D2R and Gα_i_. The SPC/E water model[34] was used, and the simulations were carried out at 310 K. In the absence of Gα_i_, no external force was applied (systems A and B); in contrast, a stabilizing force (1.0 kcal mol^-1^ Å^-2^) was applied to the N-terminal tail of the αN-helix of Gα_i_ (systems C and D2). We removed water and DOPC molecules for data analysis. The analysis of the trajectories was performed with the PTRAJ module of AMBER10[35]. Figures were prepared using PyMOL[36] and Chimera[37].

## Supporting Information

S1 Fig RMS-deviations of the simulation systems. RMSD analyses in the course of the simulation times for individual components of the systems A (A), B (B), C (C) and D (D) are shown, revealing, in general, stable simulation systems. Dopamine and D2R are fitted on the Cα-atoms of D2R, whereas Gα_i_ is fitted on the Cα-atoms of Gα_i_. For the D2R-Gα_i_ complexes (C and D), coordinates are fitted on the Cα-atoms of D2R.
(TIFF)

S2 Fig Atomic fluctuations within the simulation systems C1, C2 and D2. Atomic fluctuations for the Cα-atoms of the systems C1 (A), C2 (B) and D2 (C) are shown, which had been calculated as B-factors. The values are measured based on a fit to the Cα-atoms of the D2R-part of the complexes.
(TIFF)

S3 Fig Distances between D2R and the C-termini of Gα_i_ for the systems C1, C2 and D2. Distances between the centers of mass of D2R and the C-termini of Gα_i_ for the simulation systems C1 and C2 (A) and D2 (B) are shown.
(TIFF)

S4 Fig Overlay of average structures of the simulation systems focusing on the extracellular domains of D2R. Top view on the extracellular surface of the D2R units. For clarity, TM3 is shown in blue. The average structures are calculated after the time points concretized above. Dopamine (orange, right row) was found to stabilize extracellular receptor domains compared to the apo systems (left row), which is more pronounced at active-state D2R (bottom line). Residues of D2R (Ile183^EL2^ and Tyr408^7.35^) forming a lid over the binding pocket in system D are highlighted in red.
(TIFF)

S5 Fig Evolution of distances between TM3 and TM6 at the simulation systems E and F. The distances between the intracellular ends of TM3 and TM6 (measured as the distances between the Cα-atoms of Arg132^3.50^ and Glu368^6.30^) are shown for system E (A) and system F (B). Mean values derived from the corresponding distances at simulation systems A-D are highlighted with dashed lines. In both systems E and F, the G protein was removed. Our results indicate a higher stability of the outward movement of TM6 in the presence of dopamine.
(TIFF)

S6 Fig Atomic fluctuations and free energy of binding for dopamine at the simulation systems B and D. (A) Atomic fluctuations (calculated as B-factors after a fit on the Cα-atoms of the coordinates of D2R) for the dopamine-bound systems B and D are shown. The values above the bars represent the average fluctuation of the individual simulations 1 and 2. (B) Free energy of binding calculations were performed for dopamine-binding at the simulation systems B and D using the GBSA-Method. The numbers below the bars represent average values of each simulation system and indicate an increased binding energy of 4.8 kcal/mol in the presence of Gα_i_ (system D). The values of the individual bars are as follows (given in kcal/mol): -15.4 ± 3.9 for B1, -18.8 ± 3.9 for B2, -16.9 ± 2.8 for B3, -15.5 ± 4.1 for B4, -20.9 ± 3.0 for D1 and -22.0 ± 2.7 for D2.
(TIFF)

S7 Fig Overview of the distances between Ile183 of EL2 and Tyr408^7.35^ of TM7. The calculated distances between the side chain atoms of Ile183^EL2^ and Tyr408^7.35^ for the simulation systems A and C (A) and B and D (B) are shown. An interaction between these residues is only present in simulations of the systems D1 and D2.
(TIFF)

S8 Fig Evolution of distances between the side chains of Arg132^3.50^ and Asp350 at the simulation systems C3 to C12. The distances are highly flexible and do not offer a valid answer whether or not the ionic interaction between these residues is present at simulation system C.
(TIFF)

S1 Table Overview of all simulation systems used within this study. (DOCX)
