# Supplementary figures and images for: Comparative MD Simulations Indicate a Dual Role for Arg1323.50 in Dopamine-Dependent D2R Activation
Source: PLoS One. 2016 Jan 7;11(1):e0146612. doi: 10.1371/journal.pone.0146612 (PMC4704829; doi:10.1371/journal.pone.0146612)

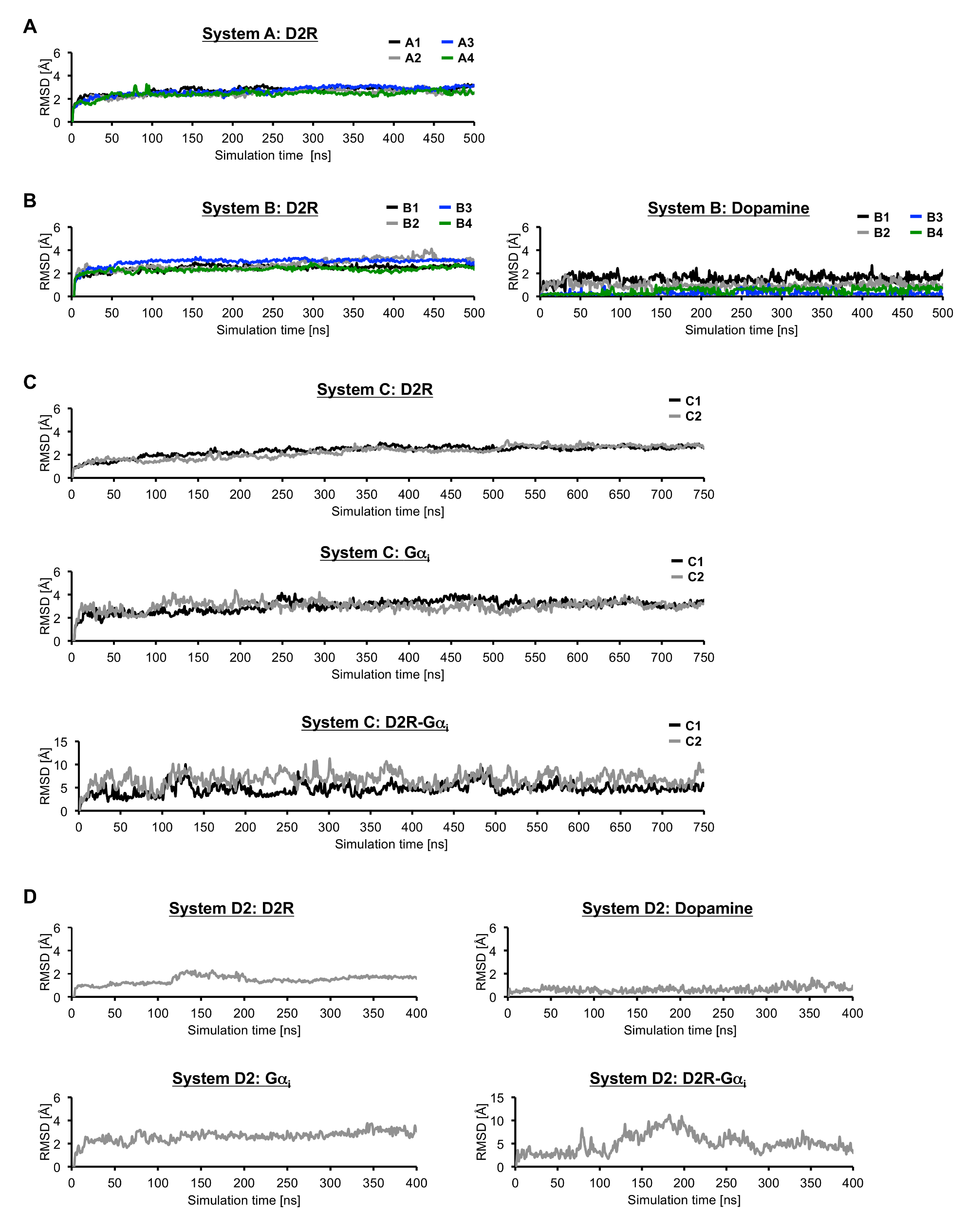

Supplement: S1 Fig — RMSD analyses in the course of the simulation times for individual components of the systems A (A), B (B), C (C) and D (D) are shown, revealing, in general, stable simulation systems. Dopamine and D2R are fitted on the Cα-atoms of D2R, whereas Gαi is fitted on the Cα-atoms of Gαi. For the D2R-Gαi complexes (C and D), coordinates are fitted on the Cα-atoms of D2R. (TIFF) [file pone.0146612.s001.tiff]

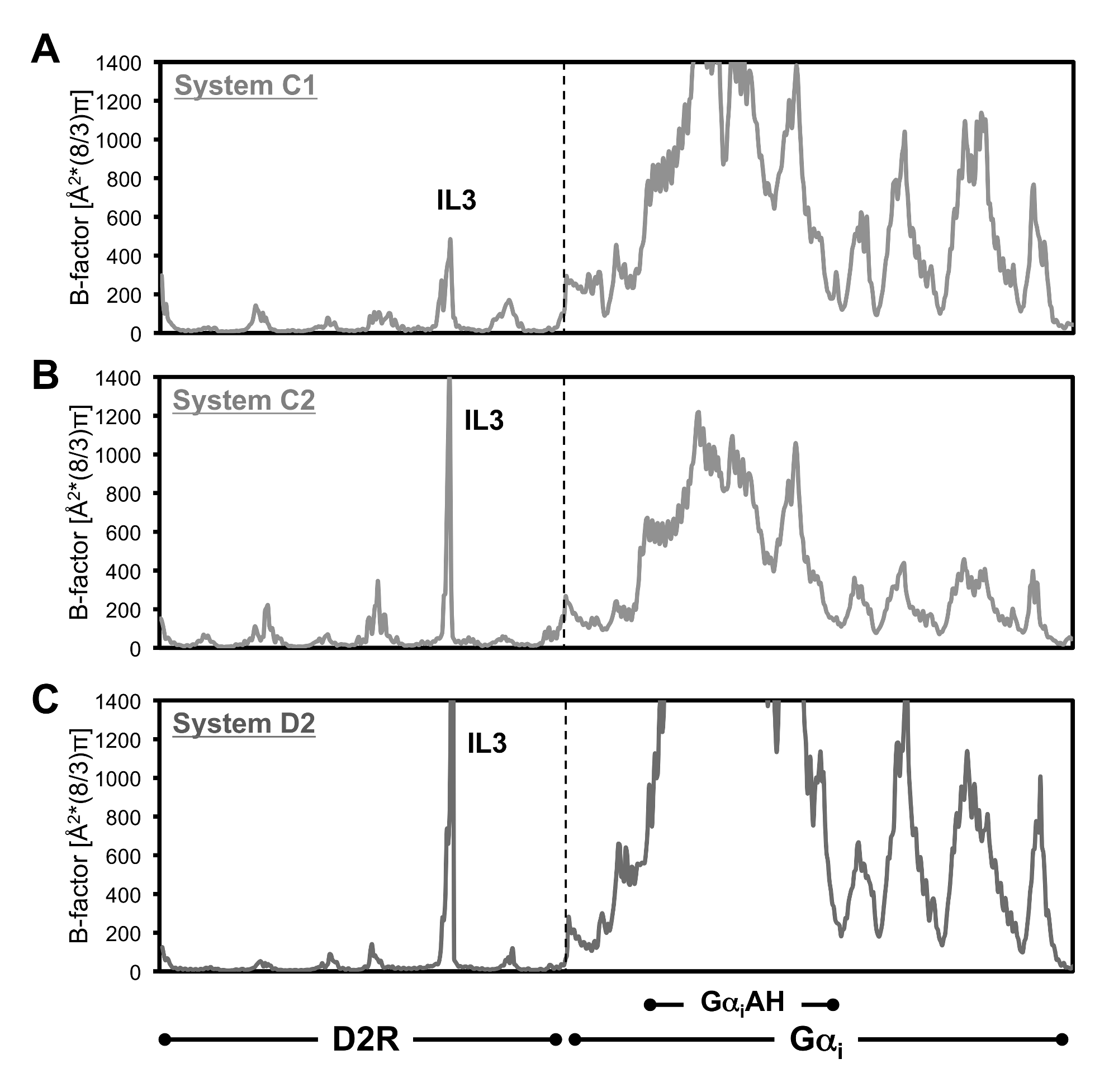

Supplement: S2 Fig — Atomic fluctuations for the Cα-atoms of the systems C1 (A), C2 (B) and D2 (C) are shown, which had been calculated as B-factors. The values are measured based on a fit to the Cα-atoms of the D2R-part of the complexes. (TIFF) [file pone.0146612.s002.tiff]

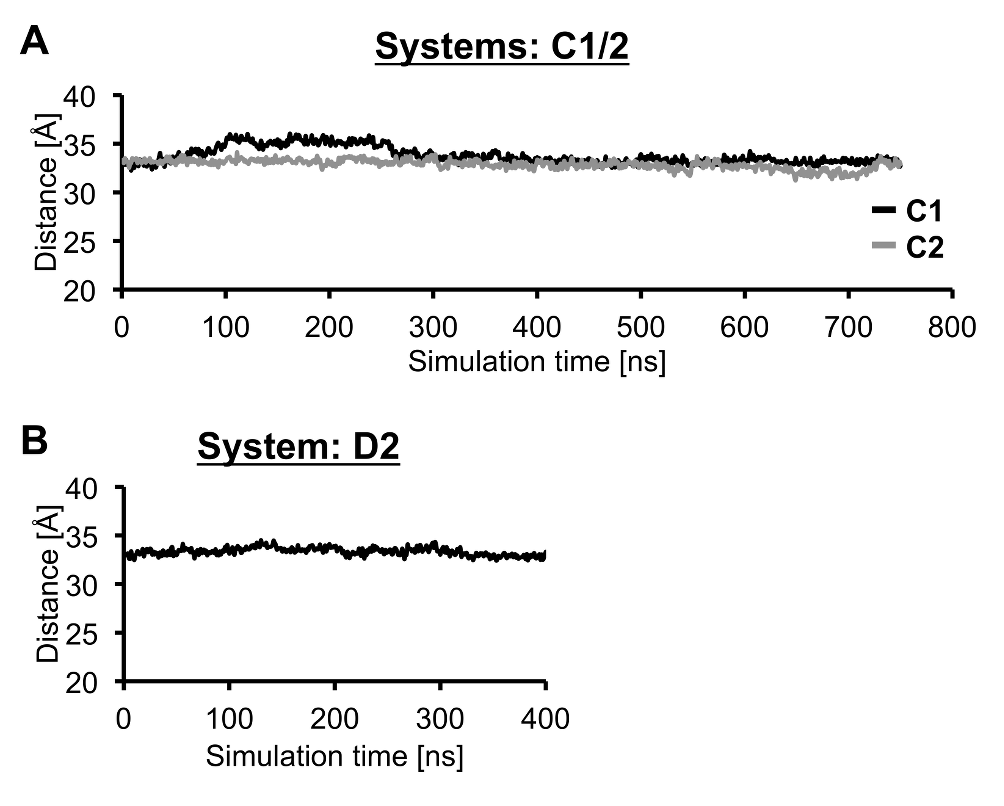

Supplement: S3 Fig — Distances between the centers of mass of D2R and the C-termini of Gαi for the simulation systems C1 and C2 (A) and D2 (B) are shown. (TIFF) [file pone.0146612.s003.tiff]

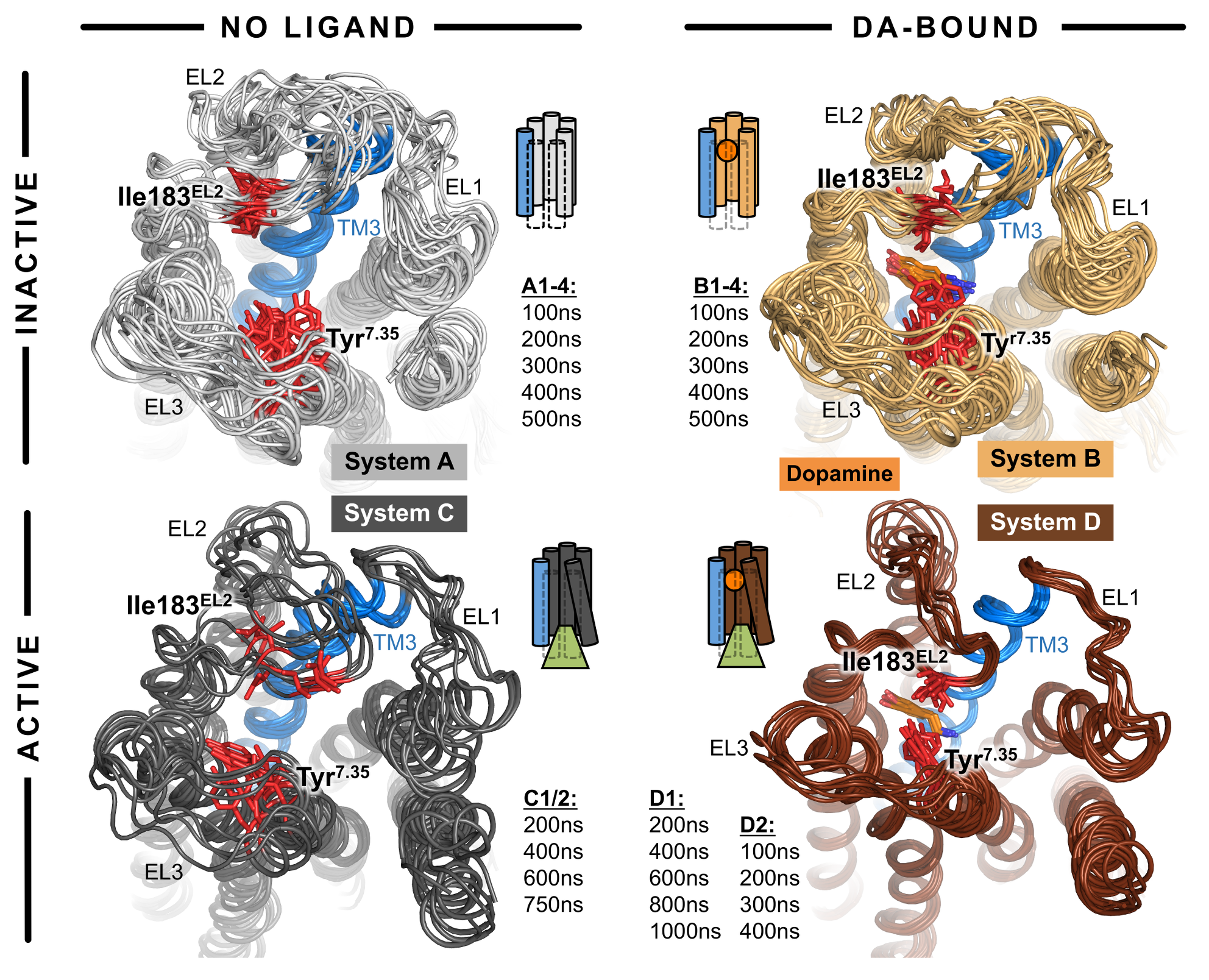

Supplement: S4 Fig — Top view on the extracellular surface of the D2R units. For clarity, TM3 is shown in blue. The average structures are calculated after the time points concretized above. Dopamine (orange, right row) was found to stabilize extracellular receptor domains compared to the apo systems (left row), which is more pronounced at active-state D2R (bottom line). Residues of D2R (Ile183EL2 and Tyr4087.35) forming a lid over the binding pocket in system D are highlighted in red. (TIFF) [file pone.0146612.s004.tiff]

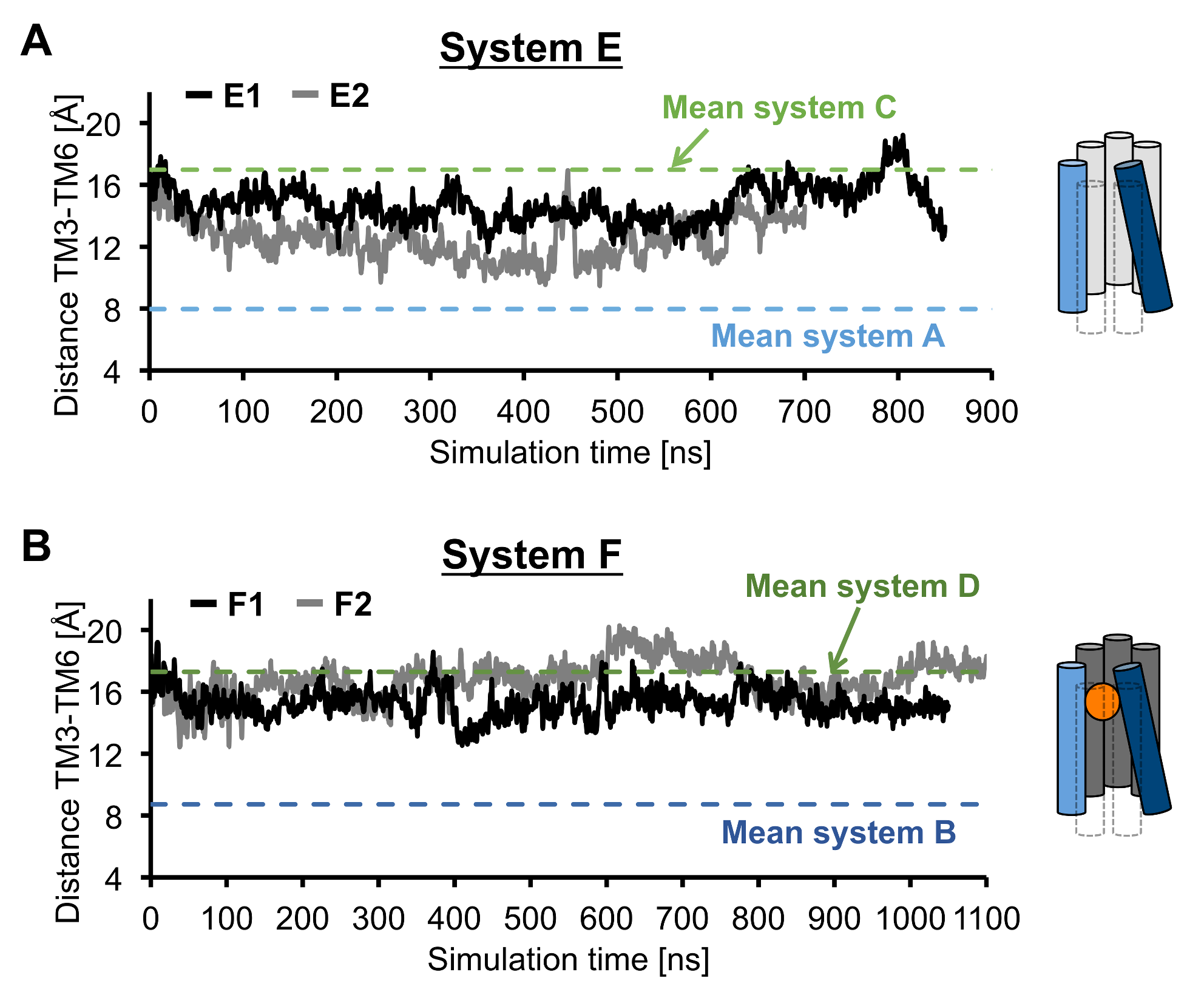

Supplement: S5 Fig — The distances between the intracellular ends of TM3 and TM6 (measured as the distances between the Cα-atoms of Arg1323.50 and Glu3686.30) are shown for system E (A) and system F (B). Mean values derived from the corresponding distances at simulation systems A-D are highlighted with dashed lines. In both systems E and F, the G protein was removed. Our results indicate a higher stability of the outward movement of TM6 in the presence of dopamine. (TIFF) [file pone.0146612.s005.tiff]

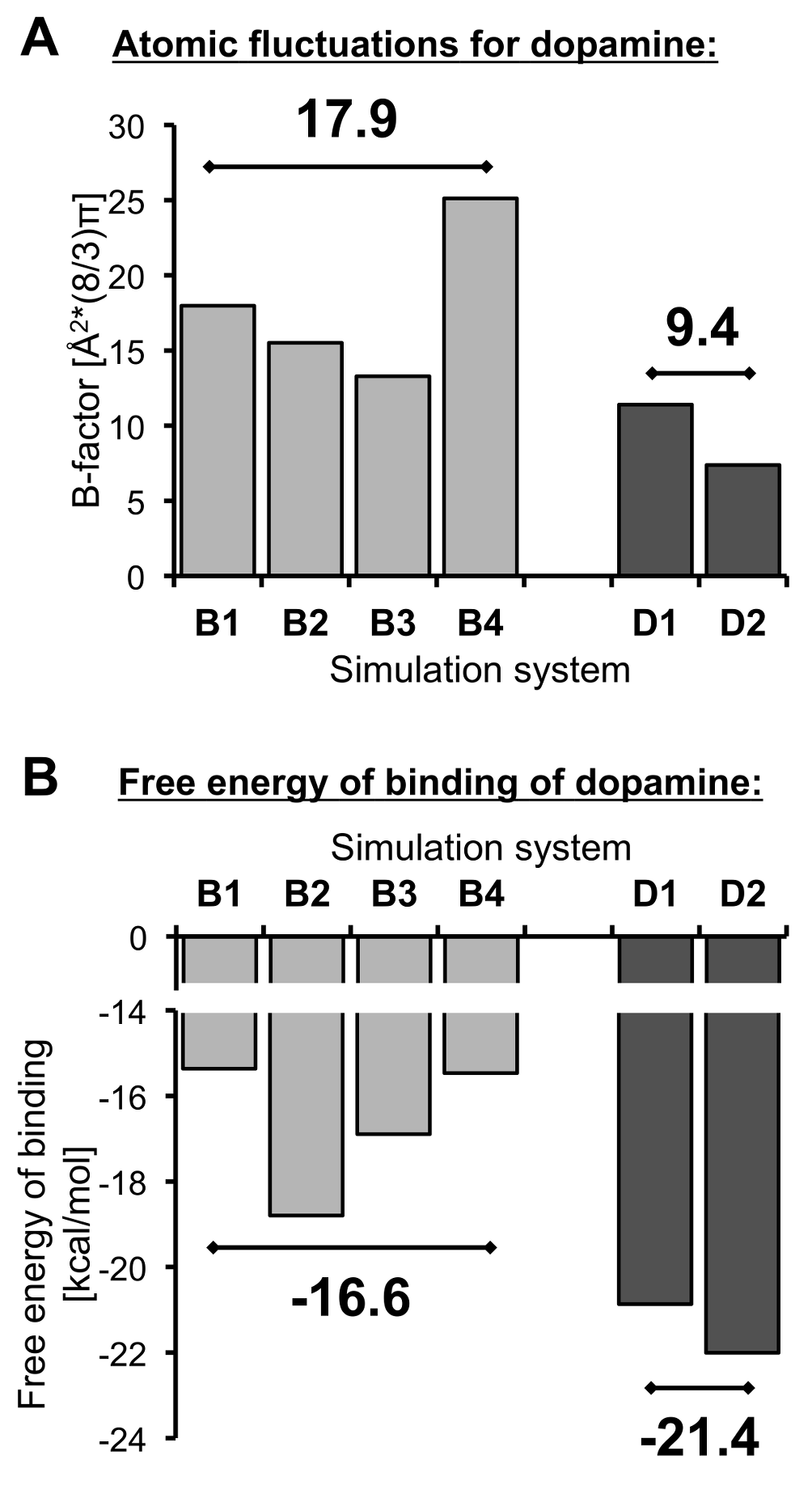

Supplement: S6 Fig — (A) Atomic fluctuations (calculated as B-factors after a fit on the Cα-atoms of the coordinates of D2R) for the dopamine-bound systems B and D are shown. The values above the bars represent the average fluctuation of the individual simulations 1 and 2. (B) Free energy of binding calculations were performed for dopamine-binding at the simulation systems B and D using the GBSA-Method. The numbers below the bars represent average values of each simulation system and indicate an increased binding energy of 4.8 kcal/mol in the presence of Gαi (system D). The values of the individual bars are as follows (given in kcal/mol): -15.4 ± 3.9 for B1, -18.8 ± 3.9 for B2, -16.9 ± 2.8 for B3, -15.5 ± 4.1 for B4, -20.9 ± 3.0 for D1 and -22.0 ± 2.7 for D2. (TIFF) [file pone.0146612.s006.tiff]

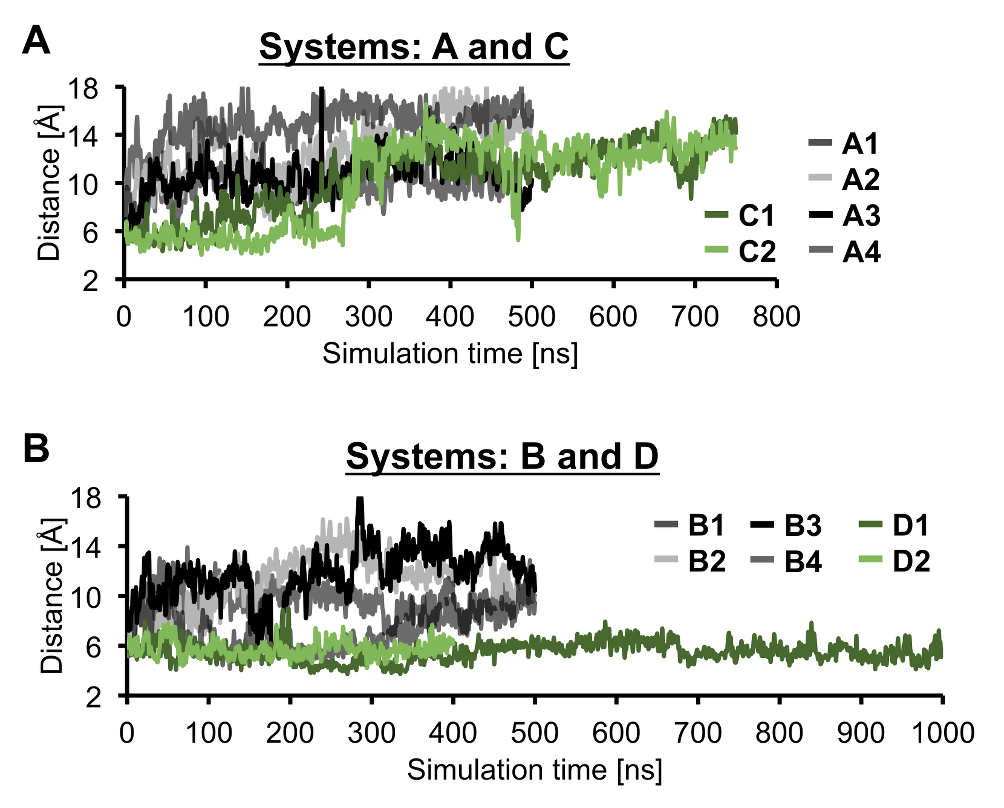

Supplement: S7 Fig — The calculated distances between the side chain atoms of Ile183EL2 and Tyr4087.35 for the simulation systems A and C (A) and B and D (B) are shown. An interaction between these residues is only present in simulations of the systems D1 and D2. (TIFF) [file pone.0146612.s007.tiff]

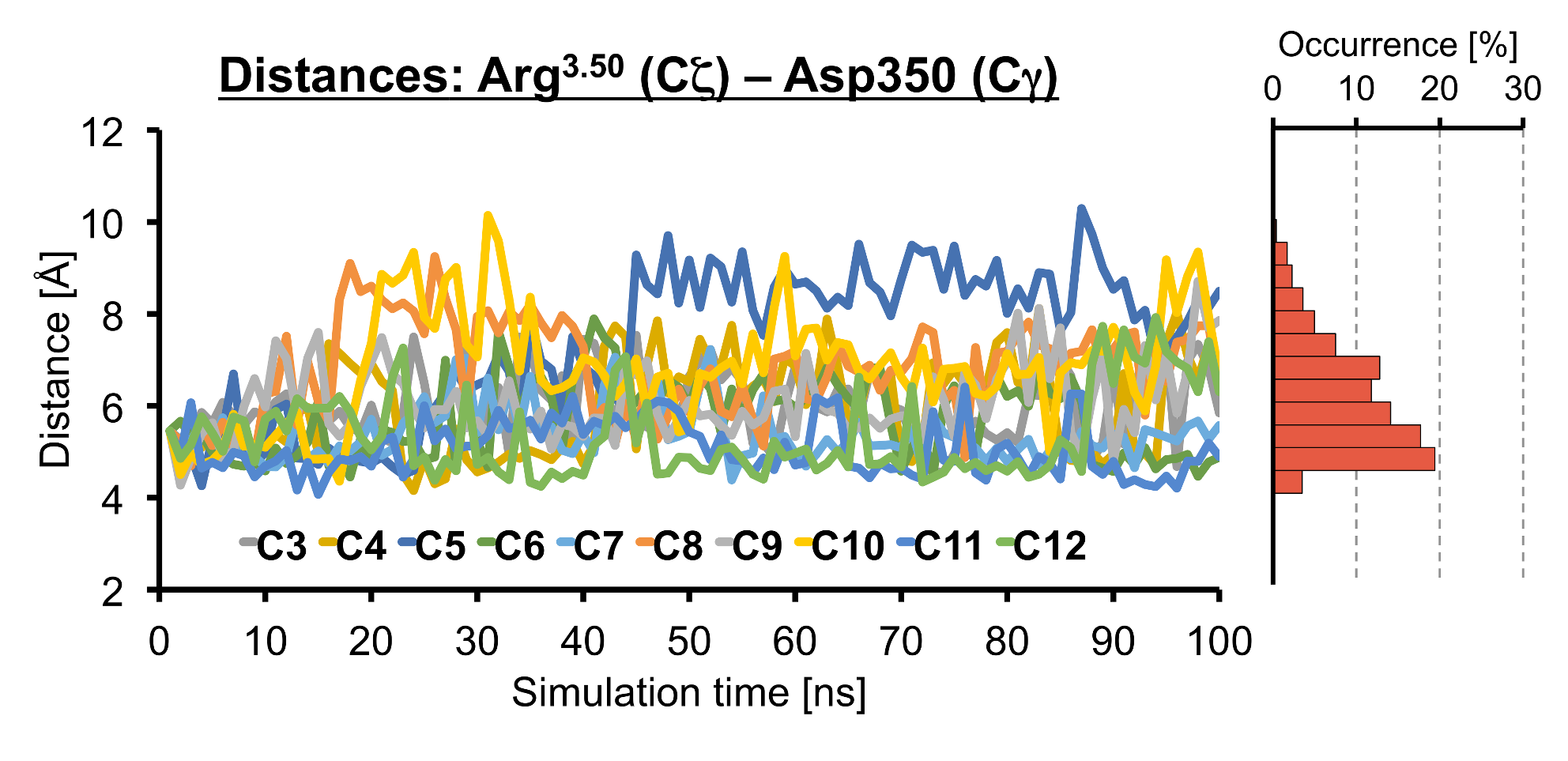

Supplement: S8 Fig — The distances are highly flexible and do not offer a valid answer whether or not the ionic interaction between these residues is present at simulation system C. (TIFF) [file pone.0146612.s008.tiff]
