# Supplementary material for: Comparative MD Simulations Indicate a Dual Role for Arg1323.50 in Dopamine-Dependent D2R Activation
Source: PLoS One. 2016 Jan 7;11(1):e0146612. doi: 10.1371/journal.pone.0146612 (PMC4704829; doi:10.1371/journal.pone.0146612)
Supplement: S1 Table — (DOCX) [file pone.0146612.s009.docx]

**Table S1.** Overview of all simulation systems used within this study.

| System | Ligand | G protein | Receptor* | Simulation times |
| --- | --- | --- | --- | --- |
| A | no | no | inactive-state | 500ns, 500ns, 500ns, 500ns |
| B | Dopamine | no | inactive-state | 500ns, 500ns, 500ns, 500ns |
| C | no | Gα_i_ | active-state | 750ns, 750ns; 10 x 100ns |
| D | Dopamine | Gα_i_ | active-state | 1000ns**, 400ns |
| E | no | no | active-state | 850ns, 700ns |
| F | Dopamine | no | active-state | 1050ns, 1100ns |

*defined as the distances between the Cα-atoms of Arg132^3.50^ and Glu368^6.30^

**previously published simulation: Kling RC, et al. (2013), PLOS One 8: e67244.
